# A summary of second systemic pulmonary shunt for congenital heart disease with pulmonary hypoxemia

**DOI:** 10.1186/s13019-020-01132-z

**Published:** 2020-05-14

**Authors:** Xue-Yong Yang, Xiao-Yong Jing, Zhe Chen, Lun Li, Xiang-Ming Fan, Jun-Wu Su

**Affiliations:** Pediatric Cardiovascular Center, Beijing Anzhen Hospital, Capital Medical University, No.2 An zhen road, Chaoyang District, Beijing, 100029 China

**Keywords:** Congenital heart disease, Primary or second systemic-pulmonary shunt, Pulmonary hypoxemia, Surgery

## Abstract

**Background:**

There has been an increasing number of children with congenital heart disease that undergo primary or second systemic-pulmonary shunt, while there are few reports on the second systemic-pulmonary shunt. Therefore, this study summarizes the experience of second systemic-pulmonary shunt for congenital heart disease in our hospital.

**Methods and results:**

Sixty-five children with congenital heart disease who underwent systemic-pulmonary shunt for the second time in our hospital were analyzed. At the early stage after the operation, cyanosis improved and SpO_2_ significantly increased. One patient died in hospital (1.54%) and the causes of death were aggravated atrioventricular regurgitation, low cardiac output syndrome, and liver failure. Early complications occurred in 18 patients (27.7%). All the children were rechecked in our hospital every 3–6 months and the McGoon index significantly increased.

**Conclusion:**

Systemic-pulmonary artery shunt can promote pulmonary vascular development, improve cyanosis symptoms, and increase the chance of radical treatment in children with pulmonary vascular dysplasia.

## Background

With the steady improvement of the comprehensive strength in medicine, congenital heart disease continues to develop in a small, difficult, and in-depth direction while operative indications continue to expand and many forbidden zones have been constantly examined in terms of systemic-pulmonary shunt [1–7]. The number of children that undergo one or more systemic-pulmonary shunt is increasing, however, there are few reports on the second systemic pulmonary shunt. In this study, we summarize the experience of the second systemic-pulmonary shunt in our hospital in the past 10 years. The details are reported as follows.

## Subjects and methods

### Subjects

From October 2010 to March 2019, 65 children with congenital heart disease who cannot be cured by one operation and thus underwent second systemic-pulmonary shunt at the Children’s Heart Center of Beijing Anzhen Hospital (597 patients completed the first systemic-pulmonary shunt in the same period) were enrolled in the present study. Among these patients, 42 patients were male and 23 patients were female. The age of these patients ranged from 4 months old to 18 years and 10 months old with an average age of 4 years and 8 months old, 25% interquartile was 12 months, median was 25 months, 75% interquartile was 6 years and 4 months. The body weight ranged within 4.5–58.2 kg with an average body weight of 15.3 kg, 25% interquartile was 9.3 kg, median was 12.9 kg, 75% interquartile was 17.5 kg. The patient’s diseases included the following: 39 patients (60%) had pulmonary atresia, in which 36 patients had a ventricular septal defect and three patients had an intact ventricular septum; 11 patients (16.9%) had tetralogy of Fallot; 9patients (13.8%) had a double outlet of the right ventricle and pulmonary artery stenosis; 3patients (4.6%) had complete transposition of the great arteries with pulmonary stenosis; and 3patients (4.6%) had single ventricle defect and pulmonary stenosis. A further 60patients (92.3%) had shunt stenosis or obstruction and 5patients (7.7%) had an excessive shunt.

### Surgical methods

#### Patients with bypass blockage or stenosis

A total of 60 patients were assigned to this group. Five of them had acute obstruction within 24 h and the remaining patients were diagnosed during the follow-up sessions. Children with acute obstruction of the systemic-pulmonary shunt channel presented with lower oxygen saturation than those before the operation and increasing oxygen uptake concentration and improving ventilation did not significantly improve oxygen saturation. The murmur of the systemic-pulmonary shunt disappeared in clinical auscultation and was diagnosed by ultrasonography. Computed tomography (CT), three-dimensional cardiovascular reconstruction or angiography was not performed. All patients underwent an emergency reoperation. General anesthesia and tracheal intubation were given and the operation within the thoracic cavity was performed through the original incision. Under cardiopulmonary bypass (CPB) or off-pump, the original shunt channel was removed, and a new shunt channel was replanted at the original shunt site. The length and direction of the shunt channel were adjusted to avoid pulling and twisting the pulmonary artery and shunt channel.

Fifty-five patients had chronic obstruction or stenosis and the children with cyanosis usually showed improvement in the early stage after the operation. In the later stage after the operation, the cyanosis was gradually aggravated and percutaneous oxygen saturation decreased. After the patient’s completed their exercise, their breathing significantly increased, activity endurance decreased, and pulmonary blood flow significantly decreased as shown on the chest X-ray films. All the patients underwent echocardiography again before the operation and 21 children underwent angiography and 35 children underwent CT three-dimensional cardiovascular reconstruction. The second operation was performed under CPB or off-pump (extracorporeal circulation was required). If the main pulmonary artery existed, the left and right pulmonary arteries were well developed and there was no obvious stenosis at the bifurcation, the pulmonary end of the second shunt channel was connected to the main pulmonary artery, the original shunt channel was removed or not removed, while the left and right proximal pulmonary artery stenosis were corrected accordingly. If the left and right pulmonary arteries were unevenly developed, the second shunt channel was usually anastomosed to the poorly developed pulmonary artery or the bilateral pulmonary arteries for the systemic-pulmonary shunt. If the first incision was a median size, the second operation could also include completing a lateral thoracotomy. Lateral thoracotomy is directed at the pulmonary artery on the dysplastic side, where the shunt channel is anastomosed between the ipsilateral pulmonary artery and the subclavian artery, which avoids entering the thoracic cavity through the original incision and free adherent tissue, reducing the possibility of massive hemorrhage. For the patients with large systemic and pulmonary collateral branches, which cannot be handled by median thoracotomy, lateral thoracotomy can often provide a beneficial counterpart to facilitate the construction of shunts while ligating large systemic and pulmonary collateral branches or fusing them into the intrinsic pulmonary artery. The larger diameter shunts are often replaced during reoperation.

### The excessive shunt group

Five patients were assigned to this group. In all patients, the disease occurred early after the operation. Cardiac echocardiography showed that the shunt was unobstructed, while X-ray chest film showed increased pulmonary blood flow, increased pulse pressure difference, and decreased diastolic pressure (even less than 30 mmHg). The patients were unable to go off the ventilator or experienced difficulties off the ventilator. Recurrent pericardial effusion or (and) pleural effusion was observed. The thoracic cavity was accessed through the original incision. In all patients, instead of removing the original shunt channel, the Gortex vessels were clamped with clips to reduce the diameter of the lumen and increase the diastolic pressure, which was mostly more than 40 mmHg. In one patient, the vessels were clamped until the diameter of the lumen reached approximately 2/3 of the normal body weight required. After the operation, the child’s circulation was still difficult to maintain and the surgeons were required to open the patient’s chest again and the shunt channel was ligated.

### Postoperative care

Routine 24-h continuous electrocardiograph (ECG) monitoring and invasive blood pressure monitoring was performed immediately after the operation in the intensive care unit. At 6 hours after the surgery, if there was no obvious bleeding, heparin was continuously infused with a rate of 2 mg/kg/24-h. Thereafter, the ventilator was removed, and aspirin was given orally at a dose of 3-5 mg/kg and the heparin was discontinued after 2 days. Due to the role of the systemic-pulmonary shunt, the pulmonary circulation blood flow increased, systemic circulation blood flow decreased, the coronary artery diastolic pressure decreased, and the relative insufficiency of cardiac blood supply led to heart failure. Through the support of ventilator therapy, cardiotonic drugs, and volume enhancement, the cardiopulmonary function was gradually adapted and recovered. Ventilation parameters were as follows: tidal volume = 8–12 mL/kg, inspiratory/expiratory = 1:1.5–2, positive end expiratory pressure = 3–5 mmHg. The below cardiotonic drugs were chosen according to patients’ conditions: dopamine 5–8 μg/kg/min, epinephrine 0.05–0.1 μg/kg/min, milrinone 0.5–1 μg/kg/min, or norepinephrine 0.05–0.1 μg/kg/min.

Firstly, the respiratory parameters were adjusted to maintain the PCO_2_ in the arterial blood gas analysis at a normal high level and the oxygen saturation was 75–85%. Secondly, adequate blood volume was ensured to keep central venous pressure (CVP) at a normal high level. The volume status was measured by CVP. Finally, cardiotonic drugs were used to maintain diastolic blood pressure close to a normal level to ensure coronary blood supply, while strengthening myocardial contractility.

### Statistical methods

Statistical analysis was conducted using statistical software SPSS19.0. Continuous data were analyzed by Shapiro-Wilktest to verify if they were normally distributed, and normally distributed measurement data were expressed as mean ± standard deviation (x ± SD) and compared using a t-test. *P* < 0.05 was considered statistically significant.

## Results

### Early postoperative results

In all children, the cyanosis symptoms were improved when compared with those before the operation, blood oxygen saturation (SpO_2_) increased from 61.5 ± 10.2% before the operation to 81.7 ± 9.2% after the operation (*P* < 0.01). The result suggests that after shunt, oxygenation was improved significantly when compared with that before shunt. The duration of the ventilator-assisted ventilation in children was 8–256 h with an average duration of 46.2 ± 21.7 h. The duration of the children’s stay in the intensive care unit (ICU) was 1–140 days with an average duration of 11.8 ± 5.2 days. During hospitalization, one patient died of an excessive shunt, the fatality rate was 1.54%, and the causes of death were aggravated atrioventricular regurgitation, low cardiac output syndrome, and liver failure. Early postoperative complications occurred in 18 patients (27.7%). Among these patients, severe low cardiac output syndrome occurred in 12 patients (66.6%), pulmonary infection occurred in 11 patients (61.1%), pleural effusion occurred in 7 patients (38.9%), pericardial effusion occurred in 6 patients (33.3%), 3 patients (16.7%) received thoracotomy again to stop bleeding, and cerebral infarction occurred in 2 patients (11.1%).

### Follow-up methods

All the children were rechecked in our hospital every 3–6 months in an outpatient follow-up period of 402 ± 330 days with a median of 450 (minimal = 90, minimal =2169) days and pulmonary artery development and patency of shunt channel was confirmed by cardiac echocardiography. The McGoon index increased from 1.08 ± 0.26 before the operation to 1.55 ± 0.48 after the operation and the difference was statistically significant (*P* < 0.01). Children with well-developed pulmonary arteries who were close to the standard for radical treatment underwent cardiac three-dimensional CT or angiography to confirm whether they met the standard for radical treatment and the situations of body-lung collateral branches in order to decide whether or not opening the chest was required again for anatomical radical resection, waiting or the reoperation of the systemic-pulmonary shunts.

## Discussion

Congenital heart disease with pulmonary blood deficiency has a special cyanosis appearance, which is easily discovered but difficult to treat. Some children cannot be cured by one operation or cannot be cured fundamentally, which seriously affects a patient’s life and is a great burden on the family and society. In 1944, Blalock et al. carried out a Blalock-Taussig systemic-pulmonary shunt for the first time; they introduced a new era for the treatment of congenital heart disease with pulmonary blood deficiency [8]. Subsequently, continuous improvements were made, and various surgical methods of systemic-pulmonary shunt have emerged. At present, central shunt and modified B-T shunt are commonly used in clinics; the majority of the shunt channels were Gortex vessels [9–12]. The initial purpose of the operation is to improve cyanosis. With the continuous improvement in understanding the disease, more attention has been paid to the role of surgery in promoting pulmonary vascular development. At present, it is not merely used to improve symptoms but mostly used as a way to promote pulmonary vascular development to create conditions for radical operations in the later stages. There are many disease types. Some can be cured by radical correction, e.g., tetralogy of Fallot, however maldevelopment of pulmonary vessels usually cannot be cured by one surgery, thus it first needs to use B-T shunt to promote the development of pulmonary vessels, and after that, the disease can be cured by surgeries. However some diseases still cannot be cured, e.g., single ventricle. In recent years, with the continuous improvement of medicine, second and multiple systemic-pulmonary shunts are increasing in children, but the operation is difficult, and the incidence of complications is high. Therefore, how to improve the skills of reoperation and reduce the complications of the second systemic-pulmonary shunt to fully play the role of the systemic-pulmonary shunt are still difficult topics in the field of cardiac surgery [13–16].

Successful thoracic opening and closing is the prerequisite for successful reoperation [17–19]. In order to prevent ventricular fibrillation during the operation, we routinely place an external defibrillation electrode plate in case a ventricular fibrillation occurred and thus defibrillation can be performed timely. Disinfection and the placement of surgical drapes were performed in the femoral artery area. If massive hemorrhage occurs during thoracotomy, emergency blood transfusion through the femoral artery can be performed and extracorporeal circulation can be established. After the first systemic-pulmonary shunt operation, the pulmonary blood increases, the heart enlarges, and the distance between the heart and the chest wall decreases. Cardiac and thoracic adhesions are also more severe, thus, a cardiac rupture can easily occur during reoperation. In order to reduce the risk of bleeding during reoperation, in sternal closure for the first operation, steel wires are often used to fix the sternum. When the chest is reopened, the wires block the space between the heart and the saw, reducing the risk of heart and large vessel ruptures. At the end of the first operation, the anti-adhesion membranes are padded on the surface of the retrosternal surface and the heart, which can effectively reduce the adhesions between the heart and surrounding tissues, reducing the chance of a heart rupture during reoperation. The order of cardiac dissection includes the aorta first since the aorta is in the front site, has a thick wall, and is obviously pulsatile. It is also easily distinguished and the chance of a rupture is low. After dissociation, the pocket line is pre-arranged for in vitro transfer. Thereafter, the right atrium is freed as the right atrium has a thin wall, severe adhesions, and an unclear boundary with surrounding tissues. The right atrium near the auricular appendage often has the most severe adhesions, thus, the separation surface should be close to the pericardial side and some pericardium is even preserved near the right atrial surface near the right atrial appendage. Attention should be paid to the protection of the phrenic nerve, which can often simplify the surgery. Although in this case, it is easy to destroy the integrity of the right pleural cavity, however, the secondary damages are relatively mild, and the phrenic nerve is not easily damaged. Extracorporeal circulation is established by right atrial intubation. After sufficient cardiac drainage under parallel circulation, the rest of the heart is freed. In this way, it can effectively reduce heart damage, especially coronary artery damage. Before completing the sternal closure, hemostasis must be performed. Before the operation, platelets and plasma are always prepared and used during the operation to make the coagulation function of the body become restored to a normal state. If active bleeding occurs after the operation, the repeated administration of hemostatic drugs can easily lead to the blockage of shunt channels with serious consequences.

Keeping the shunt channel unobstructed is the significant aim of systemic-pulmonary shunt surgery. Generally, finer shunt channels are more likely to be blocked. In this study, the diameters of the channels were selected according to the body mass: neonates: 3.5 mm; body mass 5-6 kg: 4 mm; body mass 6-10 kg: 5 mm; and body mass > 10 kg: 6 mm. Therefore, the diameter of the shunt channel cannot be chosen randomly. The closer to the aortic root that the aortic side of the shunt is, the greater the pressure difference between the two sides of the shunt channels are and the greater the shunt volume. This also means that it is easier to keep the shunt channel unobstructed, but the easer excessive shunt occurs. For example, for a 6.1 kg child, a 5 mm Gortex shunt is mostly chosen for modified BT instead of the central shunt, although the latter is easier to keep unobstructed but it has a larger shunt volume and is more difficult to be monitored after cardiac surgery. This seems to determine that the shunt location cannot be arbitrarily chosen in order to pursue a smooth rate. In fact, the most important factors for keeping the patency rate are not the tube thickness and the anastomotic location but the anastomotic technique. It is very important to keep the proper length and angle of the shunt channel and the anastomosis from bleeding. A large proportion of the systemic pulmonary shunts can be performed off-pump. The experience of the investigators is that extracorporeal circulation has no significant effect on the surgery outcome. The pulmonary artery side is clamped during the off-pump operation, making the original hypoxic situation more severe and the operation will inevitably be rushed, although, in most cases during an off-pump operation, the anastomosis can be completed but it is often challenging. In the reoperation of the systemic-pulmonary shunt, most of the operations were performed under extracorporeal circulation. Of course, if the same perfect anastomosis can be achieved, off-pump surgery is more appropriate.

Most reoperations cannot fully achieve the expected effect of systemic-pulmonary shunts due to narrow or blocked shunt channels. In this study, in 55 out of 60 children (91.7%), the disease gradually appeared during the follow-up sessions. In this study, after the first operation, although it’s not yet reached the standard of radical treatment, the children’s conditions developed well. Radical resection was performed in 10 out of 11 children (91%) during reoperation of systemic-pulmonary shunts (the replacement of the shunts of the same or larger diameter) and subsequent blockade of systemic-pulmonary collateral branches was completed. In 5 of these 10 children (50%), the pulmonary annulus developed sufficiently large and in the subsequent radical operation, the repair of the pulmonary valvular ring was not necessary, and the effect was good. In developed countries, the mainstream concept of treatment of tetralogy of Fallot is to seek an early operation and one-stop radical cure. The treatment experience of the investigators is that, for severe tetralogy of Fallot with younger and poorly developed pulmonary blood circulation, it is very difficult to treat it surgically and postoperatively even if it can be cured in one-stop. In this case, the hospitalization stay is long, the mortality rate is high, and the cost is large. The investigators tend to take a step-by-step approach, where the systemic-pulmonary shunt is first performed to promote pulmonary artery development and alleviate cyanosis and other symptoms so that the growth and development of children will not be greatly affected. Then, interventional methods such as systemic-pulmonary collateral branch occlusion, balloon dilation of the pulmonary valve, and second systemic-pulmonary shunt are performed to gain time and to gradually promote pulmonary artery development. In this method, the vast majority of children have been cured and there have been no deaths reported to date. This fully demonstrates the role of the systemic-pulmonary shunt in congenital heart disease with pulmonary blood deficiency. This also has an important impact on the improvement of reoperation skills but at present, the number of cases is small and needs further verification in the future.

The risk of an excessive systemic-pulmonary shunt is far greater than that of the insufficient systemic-pulmonary shunt. In the present study, one child died. The child was 9 years old diagnosed with single atrium, single ventricle, and severe pulmonary artery stenosis with pulmonary artery dysplasia, and the child had mild atrioventricular regurgitation and cyanosis before the operation. Suitable shunt channels and shunt locations were chosen according to his age and weight. After the first systemic-pulmonary shunt operation, the cyanosis was improved significantly and the patient was extubated 5 days after the operation, but the heart function was poor, pericardial effusion and pleural effusion repeatedly occurred, and multiple surgical drainages were performed. However, these methods did not improve the patient’s poor heart functions. Finally, it was decided that the child should undergo the reoperation. During the operation, some systemic-pulmonary shunt channels were clamped to reduce the diameter of the channels. After the operation, the child’s cardiac function improved but the patient died of physical weakness and pulmonary infection. The child was hospitalized for more than 4 months. From this painful lesson, we realize that the harmfulness of an excessive shunt is far greater than that of insufficient shunt and that children with preoperative atrioventricular regurgitation need extra caution when undergoing the systemic-pulmonary shunt. Generally, shunt channels with diameters slightly smaller than the normal standard are selected to avoid excessive shunt. Gradual aggravation of atrioventricular regurgitation is an important indicator of excessive shunt. Re-operation should be done as soon as possible; otherwise, it is easy to miss the best treatment opportunity with serious consequences.

After the operation of the systemic-pulmonary shunt, maintaining cardiopulmonary function, preventing and treating low cardiac output syndrome, and gradually adapting cardiopulmonary function are the first and foremost tasks to be completed, which are related to the success or failure of the systemic-pulmonary shunt. The essence of the systemic-pulmonary shunt is to inject some blood of systemic circulation into the pulmonary circulation by high pressure through the shunt channels, which will inevitably lead to the corresponding increase of pulmonary circulation blood volume and left ventricular return blood volume. The result is a sudden increase in the left ventricular volume load, with relatively insufficient coronary artery blood supply caused by the decrease of systemic circulation diastolic blood pressure caused by the shunt and easily causes left heart failure. Although the improvement in oxygen supply is due to the increase in blood oxygen saturation, fine adjustments are still needed to avoid drastic changes in the circulation and only then the body can gradually adapt to the adjustments. First, blood volume needs to be replenished: especially in children undergoing off-pump systemic-pulmonary shunt as there is no process of adjusting blood volume by extracorporeal circulation. The volume should be replenished in time during and after the operation to accommodate the sudden increase in volume requirements. However, it is difficult to accurately estimate this volume requirement due to individual differences, with the central venous pressure reaching a normal high level as the standard. In terms of the rational use of cardiotonic drugs, a sudden increase in cardiac volume load will inevitably impact cardiac function, especially in children whose cardiac function is marginalized before the operation, thus, the rational use of cardiotonic drugs is very important. Vasoactive drugs such as dopamine, epinephrine, and norepinephrine are routinely used during and after the operation to help reduce further illness and mortality in children. In terms of the rational use of ventilators, the proper ratio of volumes of pulmonary circulation to systemic circulation can be adjusted by ventilator parameters. Overall, the circulation in children undergoing systemic-pulmonary shunt is mostly single ventricular physiology and the systemic-pulmonary shunt channel is an important channel for the blood flow adjustment between the two. When the diameter of the channel is constant, the resistance between the two circulations determines the size of the shunt. When the oxygen saturation is between 80 and 85%, the blood flow ratio of systemic circulation to pulmonary circulation is close to 1:1. When oxygen saturation is too high, it is likely to become excessive shunt and often not necessarily beneficial to the child. Initially, after systemic-pulmonary shunt, pulmonary artery pressure increases as a result of the massive blood flow to the pulmonary circulation. Evidently, effective filtration pressure of tissue fluid equates to (capillary blood pressure + colloidal osmotic pressure of tissue fluid) - (hydrostatic pressure of tissue fluid + colloidal osmotic pressure of plasma). If other conditions remain unchanged, an increase in the capillary filtration pressure in the lung inevitably leads to an increase in tissue fluid production in the lung and the chance of pulmonary edema increases to affect oxygenation. This is the main reason why blood oxygen saturation increases and decreases immediately after a systemic-pulmonary shunt. In order to prevent sudden sharp changes in pulmonary blood flow, it is very important to set up reasonable ventilator parameters. In this study, the partial pressure of PCO_2_ in blood gas analysis was usually adjusted to 40-45 mmHg, the SpO_2_ was maintained at 80–85%, for children with cardiac dysfunction, the partial pressure of PCO_2_ in blood gas analysis was adjusted to 45-50 mmHg, and the SpO_2_ was maintained at 75–80% and can even be as low as 70–75% to keep lactate at a normal level to avoid the sudden increase of pulmonary blood flow that causes a serious impact on cardiopulmonary function while allowing a gradual adaptation in the body. It is important to note that, for children undergoing systemic-pulmonary shunt, careful attention should be paid to sputum suction and hand-controlled breathing and excessive ventilation should be avoided because high oxygen and low carbon dioxide can dramatically increase the lung blood volume, causing drastic and dangerous fluctuations in the circulation. The limitation of this study is that we did not collect the pro-thrombotic factors (genetic, epigenetic), which should be addressed in future studies.

## Conclusion

In summary, systemic-pulmonary artery shunt can promote pulmonary vascular development, improve cyanosis symptoms, and increase the chance of radical treatment in children with congenital heart disease, pulmonary blood deficiency, and pulmonary vascular dysplasia. With the continuous improvement of skills in the reoperation process, second systemic-pulmonary shunt is safe and feasible. While ensuring the patency of the shunt channel, early intensive monitoring and comprehensive treatment after the operation ensures successful surgery outcomes.

## Data Availability

The datasets used and/or analysed during the current study available from the corresponding author on reasonable request.

## Abbreviations

CT: Computed tomography
CPB: Cardiopulmonary bypass
ECG: Electrocardiograph
CVP: central venous pressure
x ± SD: mean ± standard deviation
ICU: intensive care unit
